# Reprojection Error Analysis and Algorithm Optimization of Hand–Eye Calibration for Manipulator System

**DOI:** 10.3390/s24010113

**Published:** 2023-12-25

**Authors:** Gang Peng, Zhenyu Ren, Qiang Gao, Zhun Fan

**Affiliations:** 1 School of Artificial Intelligence and Automation, Huazhong University of Science and Technology, Wuhan 430074, China; penggang@hust.edu.cn (G.P.); 13781366835@163.com (Z.R.); 2 Key Laboratory of Image Processing and Intelligent Control, Ministry of Education, Wuhan 430074, China; 3 College of Engineering, Shantou University, Shantou 515063, China

**Keywords:** hand–eye calibration, reprojection error analysis, manipulator object grasping

## Abstract

The Euclidean distance error of calibration results cannot be calculated during the hand–eye calibration process of a manipulator because the true values of the hand–eye conversion matrix cannot be obtained. In this study, a new method for error analysis and algorithm optimization is presented. An error analysis of the method is carried out using a priori knowledge that the location of the augmented reality markers is fixed during the calibration process. The coordinates of the AR marker center point are reprojected onto the pixel coordinate system and then compared with the true pixel coordinates of the AR marker center point obtained by corner detection or manual labeling to obtain the Euclidean distance between the two coordinates as the basis for the error analysis. We then fine-tune the results of the hand–eye calibration algorithm to obtain the smallest reprojection error, thereby obtaining higher-precision calibration results. The experimental results show that, compared with the Tsai–Lenz algorithm, the optimized algorithm in this study reduces the average reprojection error by 44.43% and the average visual positioning error by 50.63%. Therefore, the proposed optimization method can significantly improve the accuracy of hand–eye calibration results.

## 1. Introduction

With the development of robotics and sensor technology [1,2,3,4], robotic grasping has gradually become a significant function for robots. It involves identifying and locating a target object through a visual sensor. Therefore, many sensor calibration methods [5,6] have emerged, including hand–eye calibration algorithms [7]. To ensure the accuracy of visual information and to achieve coordinated hand–eye motion, it is essential to analyze the manipulator’s hand–eye visual calibration problem and to improve its accuracy. Hand–eye calibration establishes a transformation matrix between the pixel coordinate system of the camera and spatial coordinate system of the manipulator. By transforming the pixel coordinates into the coordinate system of the manipulator, the robot can calculate the motor movements necessary to reach the target position and to control the manipulator. Hand–eye calibration can be divided into eye-to-hand and eye-in-hand calibrations, depending on the camera installation position. This study focuses on experiments using an eye-in-hand installation.

Traditional calibration methods build calibration models based on preset imaging scenes and select optimal algorithms, including reference-based, active vision, and self-calibration methods, to calculate camera parameters based on scene geometric constraints. Based on the reference object camera calibration method, the corner points of the target image were extracted as control points, a system of equations corresponding to the pixels and spatial coordinates was established, and an optimization algorithm was used to calculate the parameters in which the shape and size of the reference object and other information were known. The reference object is divided into one-dimensional straight lines [8], two-dimensional plane calibration plates, and three-dimensional solid blocks, according to the spatial dimension. Because of their simple production and controllable accuracy, flat calibration plates are often used as targets in industrial applications instead of calibration blocks. Commonly used calibration-plate patterns include checkerboards, solid circles [9], and concentric rings. Recently, various templates have been proposed for this purpose [10]. The Zhengyou calibration method [11], based on a chessboard calibration board, is a classic representative of this type of method. This method has strong imaging constraints, a simple calibration process, and a high algorithm robustness. This type of method has strong imaging constraints, a simple calibration process, and high algorithm robustness; however, the production and maintenance cost of high-precision reference objects is high, and it is lost in situations where it is impossible to carry the significance of the reference objects. Camera calibration methods based on active vision obtain multiple images by accurately controlling special movements, such as the pure rotation and translation of the camera or target, and use controllable quantitative motion constraints to determine the internal and external information of the camera. This is an important aspect of self-calibration methods. Typical methods include calibrations based on pure rotational motion [12], three orthogonal translation motions, plane orthogonal motion, an infinite plane homography matrix, hand–eye calibration [13], and self-calibration based on projection reconstruction. Active visual calibration technology can linearly solve internal camera parameters with strong algorithm robustness. However, strict requirements for the control equipment limit their use and promotion. The camera self-calibration method does not require the setting up of a reference object or controlling its precise movement. It only uses the geometric consistency constraints of corresponding points in multiple image frames [14,15] to solve the basic camera matrix and does not rely on scene structure and motion information, including directly solving the Kruppa equation, methods based on absolute quadratic curves and absolute quadric surfaces [16,17], Pollefeys module-constrained calibration [18,19], and hierarchical stepwise calibration methods under variable internal parameters [20].

The focus of this study was a hand–eye calibration algorithm. Since 1989, Shiu [21] and Tscai [7] presented the hand–eye calibration problem. Domestic and foreign scholars have conducted considerable research. For example, Chen et al. proposed a noise-tolerant algorithm for robot sensor calibration using a planar disk in any three-dimensional direction [22]. Li et al. proposed a hand–eye calibration method for linear laser sensors based on three-dimensional reconstruction [23]. Zhang et al. proposed a calibration method of a hand–eye system with rotation and translation coupling [24]. The solutions to hand–eye calibration problems can be divided into two categories according to the solution order of the calibration matrix. The first solves the rotation and translation vectors of the matrix simultaneously. A typical algorithm is Andreff ‘s closed-loop solution of the hand–eye calibration equation based on the matrix direct product for the calibration of small-scale moving measurement scenarios [25]. Tabb proposed a hand–eye calibration algorithm based on an iterative optimization method and solved it using a nonlinear optimizer [26]. Jiang et al. proposed a method to calibrate the hand–eye of the EOD robot by solving the AXB = YCZD problem [27]. The second solves the rotation matrix of the calibration matrix first and then the translation vector of the calibration matrix. The most common method is the method proposed by Tsai and Lenz [7], which introduces the rotation axis–rotation angle system to describe the rotation motion. Liu et al. proposed a hand–eye calibration method for a robot vision measurement system [28]. Zou et al. performed a hand–eye calibration on arc welding robots and laser vision sensors through semi-definite programming [29]. Deng et al. proposed a hand–eye calibration method based on a monocular robot [30]; however, the above algorithm still has some space for optimization.

In the hand–eye calibration process of a real manipulator, the actual error of the calibration result cannot be calculated because the true value of the hand–eye transformation matrix cannot be obtained. Therefore, a new error analysis method is required. A reprojection error analysis method was proposed. The contribution of this study is to determine the reason for certain errors in each calibration algorithm; they ignore the calculation errors in the transformation matrix of the augmented reality (AR) marker coordinate system relative to the camera coordinate system, Tmc , making it difficult to obtain high-precision calibration results. The method proposed in this study innovatively utilizes the prior knowledge that the position marked by the AR is fixed in the calibration process to conduct error analysis and algorithm optimization. First, the coordinates of the center point of the AR mark are reprojected to the pixel coordinate system and are then compared with the real pixel coordinates of the center point of the AR mark obtained by corner detection or manual labeling to obtain the Euclidean distance between the two coordinates, which is the basis for error analysis. Finally, we fine-tuned the results of the hand–eye calibration algorithm to obtain the smallest reprojection error, thereby obtaining high-precision calibration results.

## 2. Coordinate System Definitions and Hand–Eye Calibration Equation

### 2.1. Coordinate System Definitions

In the robot arm hand–eye vision grasping system, the visual information obtained through the camera is described in the camera coordinate system. However, the reference coordinate system for robot arm motion planning is the robot arm base coordinate system. Therefore, to realize the vision of a robot arm, grasping usually requires converting the visual information into a robot arm base coordinate system for description. Let Teb  be the transformation matrix of the robot arm end coordinate system relative to the base coordinate system and Tce  be the transformation matrix of the camera coordinate system relative to the robot arm end coordinate system. Then, the transformation matrix of the camera coordinate system relative to the base coordinate system of the robotic arm is Teb =Teb ·Tce . Among them, Teb  can be calculated using the joint angles of the robotic arm and the forward kinematics equation, whereas the matrix Tce  is solved through the hand–eye calibration algorithm.

**Figure 1:**
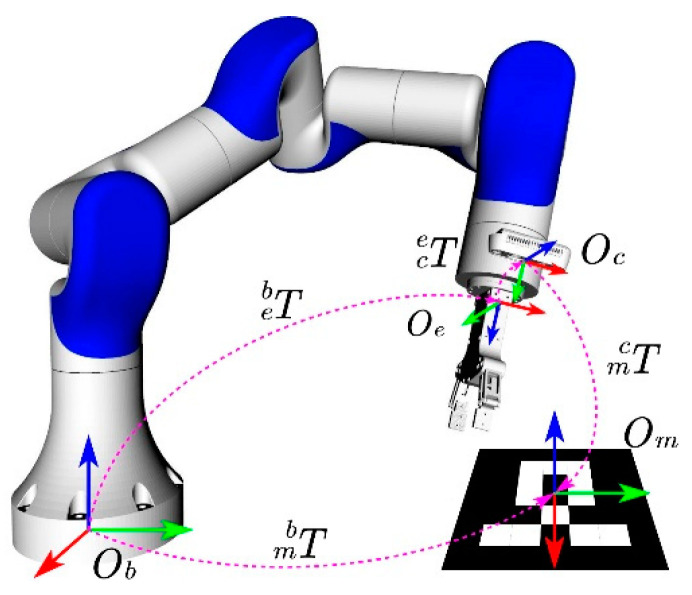
Coordinate system and transformation relationship diagram in the process of hand–eye calibration. The coordinate systems for the manipulator base, manipulator end, camera, and AR marker are defined as $O_{b}$, $O_{e}$, $O_{c}$, and $O_{m}$. In the coordinate system in the figure, the red arrow represents the x-axis, the green arrow represents the y-axis, and the blue arrow represents the z-axis.

To solve the above hand–eye calibration problem, it is necessary to use a calibration object, which requires the attitude of the calibration object coordinate system relative to the camera coordinate system to be calculated in real time. AR markers were used in this study. The camera for the robotic arm visual grasping system was installed in an eye-in-hand manner, and the origins of the manipulator base, manipulator end, camera, and AR marker coordinate systems are defined as $O_{b}$, $O_{e}$, $O_{c}$, and $O_{m}$, respectively. The coordinate systems and their transformation relationships during the hand–eye calibration process are shown in Figure 1.

Let Tmc be the transformation matrix of the AR marker coordinate system relative to the camera coordinate system. According to Figure 1, the transformation relationship between the AR marker coordinate system and base coordinate system of the manipulator Tmb is

(1) Tmb=T eb · T · Tmcce

### 2.2. Hand–Eye Calibration Equation

In Equation (1), Tce is fixed, which is the hand–eye transformation matrix to be solved. If the position of the AR marker relative to the base coordinate system of the manipulator is constant, Tmb is fixed. For a certain state $S_{i}|i\in N$ of the manipulator, Tmci can be calculated using the size of the AR marker, the corner coordinates, and the internal parameters of the camera. Therefore, for a certain state $S_{i}$ of the manipulator, the above equation can be expressed as:

(2) Tmb=T eb · T · Tmcice, i∈N

There are two fixed unknown matrices, Tmb and Tce, in the above equation: to solve these two unknown matrices, it is necessary to control the manipulator to move in two different states, and the position of the AR marker should remain unchanged during movement. Using these two states, the following equations were obtained:

(3)  Tmb=T eb ·T · Tmc1ce Tmb=T eb ·T · Tmc2ce

According to the above Equation, the following can be obtained

(4) T eb · T · Tmc1ce=Tmb=T eb · T · Tmc2ce

The above Equation can be converted to


(5) $$\frac{{}_{e}^{b}T_{2}^{-1}{\cdot \;}_{e}^{b}T_{1}}{A}\cdot \frac{{}_{c}^{e}T}{X}=\frac{{}_{c}^{e}T}{X}\cdot \frac{{}_{m}^{c}T_{2}{\cdot \;}_{m}^{c}T_{1}^{-1}}{B}$$

Furthermore, the problem of solving the hand–eye transformation matrix Tce is transformed into a problem of solving the homogeneous Equation AX = XB, where A, X, and B are 4 × 4 homogeneous transformation matrices.

To further solve the homogeneous equation AX = XB, the homogeneous transformation matrix in Equation (5) is expressed in the form of a rotation matrix and translation vector as follows:


(6) $$\frac{\left[\begin{array}{cc} R_{A} & t_{A}\\ 0 & 1 \end{array}\right]}{A}\frac{\left[\begin{array}{cc} R & t\\ 0 & 1 \end{array}\right]}{X}=\frac{\left[\begin{array}{cc} R & t\\ 0 & 1 \end{array}\right]}{X}\frac{\left[\begin{array}{cc} R_{B} & t_{B}\\ 0 & 1 \end{array}\right]}{B}$$

By expanding the above formula, the equations to be solved can be obtained as

(7) $$\left\{\begin{array}{c} R_{A}\cdot \;R=R\;\cdot \;R_{B}\\ \left(R_{A}-I\right)t=R\cdot t_{B}-t_{A} \end{array}\right.$$

In the above Equation, $R_{A}$, $R_{B}$, $t_{A}$, and $t_{B}$ can be measured, and I is the unit matrix. There are many solutions for obtaining rotation matrix R and translation vector t from this equation set, such as the Tsai–Lenz algorithm [7] and the Horaud [31], Andreff [25], and Daniilidis [32] algorithms. Subsequently, we conducted simulation experiments and developed an error analysis method to analyze and optimize the above four hand–eye calibration algorithms.

## 3. Reprojection Error Analysis Method of Calibration Algorithms

### 3.1. Hand–Eye Calibration Algorithm Simulation Experiments

**Figure 2:**
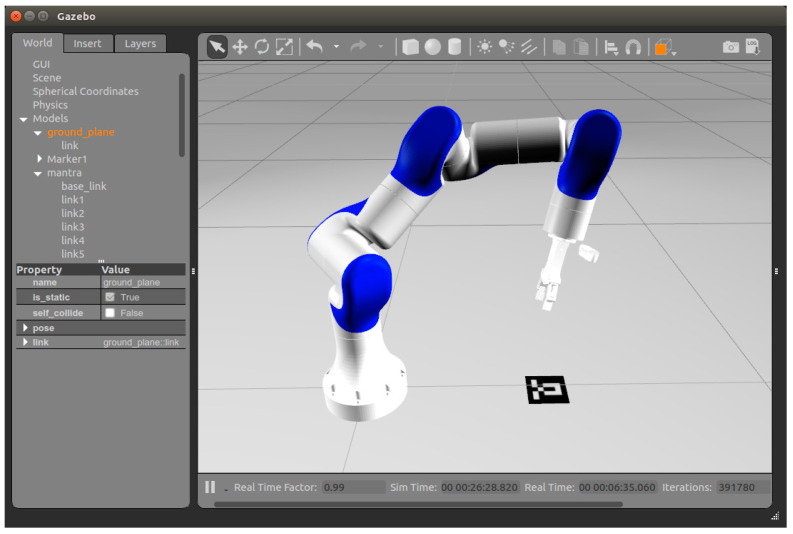
Hand–eye calibration simulation experimental environment built in ROS and Gazebo simulation platforms. The resolution of the RGB camera in the test environment is 640 × 480 pixels and the size of the AR mark is 10 cm × 10 cm.

**Figure 3:**
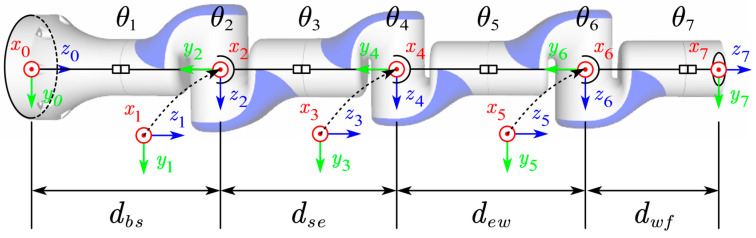
Establishment of the coordinate system of the 7-DOF manipulator in this article. In the coordinate system in the figure, the red arrow represents the x-axis, the green arrow represents the y-axis, and the blue arrow represents the z-axis.

In this study, the ROS and Gazebo simulation platforms were used to build the simulation environment shown in Figure 2 to test the performance of the above four hand–eye calibration algorithms [7,25,31,32]. With reference to the manipulator employed in the practical experiment, the simulation employed the standard DH parameter method to conduct the kinematic modeling of the 7-degree-of-freedom manipulator. The link coordinate system of the manipulator was established as illustrated in Figure 3, and the respective DH parameters are listed in Table 1. The resolution of the RGB camera in the test environment was 640 × 480 pixels, and Gaussian noise with a mean value of 0 and a variance of 0.07 was added to the collected images. The internal parameters are Equations (19) and (20), which are the internal camera parameters used in the real experiment. In the simulation environment, the size of the AR marks was 10 × 10 cm. The true values of Tce and Tmb for the simulation experiments are listed in Table 2.

**Figure 4:**
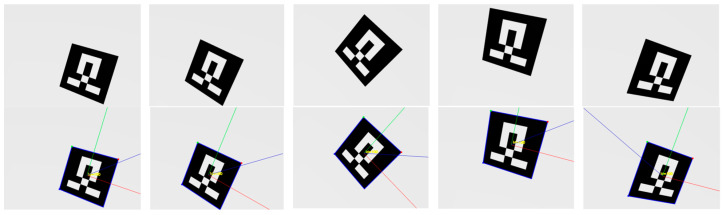
AR marker images and corner detection results collected in the simulation environment.

In the simulation experiment, 22 different states of the robotic arm were manually set to ensure that the center point information of the AR mark could be detected in each state and that the joint angles of the robotic arm changed significantly between each state. Some of the collected images and AR marker center point detection results are shown in Figure 4. The calculation results for each calibration algorithm are listed in Table 3.

To quantitatively evaluate the performance of each calibration algorithm, the translation error ${err}_{t}$ of the hand–eye transformation matrix was defined as the two norms of the difference between the calculated value $t_{c}$ of the translation vector and the true value $t_{r}$. The translation error was measured using the following Euclidean distance:

(8) $${\;err}_{t}={\left\|t_{c}-t_{r}\right\|}_{2}$$

Similarly, the rotation matrix R is first converted to a Euler angle E, and the Euler angle is expressed in vector form as $E={\left[roll,pitch,yaw\right]}^{T}$, and then the rotation error ${err}_{R}$ of the hand–eye conversion matrix can be defined as:

(9) $${\;err}_{R}={\left\|E_{c}-E_{r}\right\|}_{2}$$

**Figure 5:**
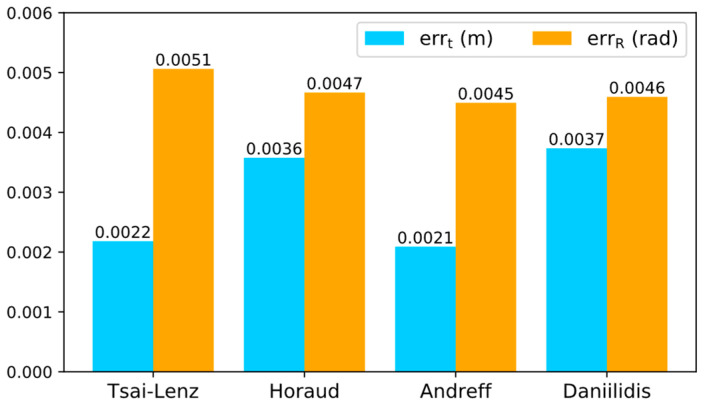
Euclidean distance error statistics of each calibration algorithm (according to the real values of the parameters in the simulation environment in Table 2). The statistical figure reveals that the translation error in the hand–eye conversion matrix, as computed by the Tsai–Lenz and Andreff algorithms, is notably lower compared to the other two algorithms. However, it is noteworthy that the rotation error in the hand–eye conversion matrix, calculated using the Tsai–Lenz algorithm, is marginally higher than in the other algorithms.

According to the real values of the parameters in the simulation environment, the statistical results of the translation and rotation errors of the hand–eye transformation matrix calculated by each calibration algorithm are shown in Figure 5. It can be observed from the statistical figure that the translation error of the hand–eye conversion matrix calculated by the Tsai–Lenz and Andreff algorithms is significantly lower than that of the other two algorithms; however, the rotation error of the hand–eye conversion matrix calculated by the Tsai–Lenz algorithm is slightly higher than that of the other algorithms. Overall, the calibration accuracies of the Tsai–Lenz and Andreff algorithms are relatively high in the simulation environment.

### 3.2. Heuristic Error Analysis

As shown in Figure 5, each algorithm has a certain optimization space. The following presents a heuristic error analysis of the simulation results. During the eye-in-hand calibration process, the position of the calibration object is fixed relative to the base coordinate system of the manipulator. Therefore, in theory, Tmb should be a fixed value; however, according to the coordinate transformation relationship shown in Figure 1, after calculating the hand–eye transformation matrix Tce, Tmb can be calculated by the following equation

(10) Tmb=T eb  · T ce · T mc 

In the process of hand–eye calibration, it is necessary to collect multiple datasets; hence, the set C=Tmbi,i∈N can be calculated. Because Tmb, calculated by the above formula, will be affected by the parameter error in Tce, a simple conjecture can be established. If the fluctuation degree of the data in set C is smaller, the errors of Tce are smaller.

**Figure 6:**
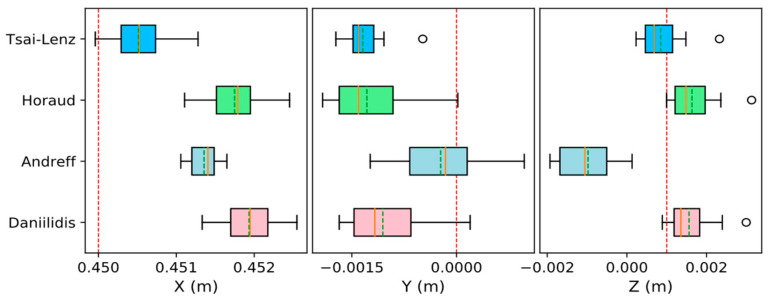
Visualization of fluctuation range of parameters in the translation matrix of Tmbi. In the illustration, the authentic data are represented by the red dotted line, whereas the orange solid line and green dotted line correspond to the median and average values derived from each algorithm, respectively. The left and right sides of the rectangle signify the lower and upper quartiles, respectively. The extent of the I-shaped area conveys the fluctuation range of the data, and the outliers are denoted by open circles.

To facilitate the analysis of the degree of data fluctuation, box plots were used to visualize the fluctuation range of each parameter of the translation matrix $t_{i}$ in Tmbi, and the results are shown in Figure 6. The red dotted line in the figure represents the real value, whereas the orange solid and green dotted lines represent the median and average of the data calculated using each algorithm, respectively. The left/right sides of the rectangle are the lower/upper quartiles, respectively; the size of the I-shaped area reflects the fluctuation range of the data; and the open circles represent the outliers.

The corresponding data fluctuation range of the Tsai–Lenz algorithm is small, which is in line with expectations. However, the Andreff algorithm has a large fluctuation range for the corresponding data, which is inconsistent with expectations. Therefore, it is unreasonable to determine the error of the hand–eye conversion matrix Tce by the fluctuation degree of the data in set C because the above conjecture ignores the influence of the error of Tmc on the calculation result of Tmb.

### 3.3. Reprojection Error Analysis

From the results of the heuristic error analysis, it can be observed that Tce and Tmc may have errors; therefore, it is unreasonable to calculate Tmb using Equation (10). Because the position of the AR marker is fixed during the calibration process, the following error analysis process assumes that Tmb is known and fixed.

According to the coordinate transformation relationship shown in Figure 1, the pose representation of the AR marker in the camera coordinate system can be obtained as follows:

(11) Tmc=T ec  · T be · T mb 

If the AR marker coordinate system $\left\{O_{marker}\right\}$ is defined as the world coordinate system, then Twc=Tmc. According to the pinhole camera imaging model, the conversion relationship between the coordinates $\left(X_{w},Y_{w},Z_{w}\right)$, the pixel coordinates $\left(u,v\right)$ in the AR marker coordinate system and the *z*-axis coordinate $Z_{c}$ in the camera coordinate system can be obtained as follows:

(12)  Zcuv1=MTwcXwYwZw1=MTmcXmYmZm1=MTecTbeTmbXmYmZm1

Based on the above definition, the coordinates of the origin $O_{marker}$ of the AR marker coordinate system in the world coordinate system $\left(X_{w},Y_{w},Z_{w}\right)=\left(X_{m},Y_{m},Z_{m}\right)=\left(0,0,0\right)$, and the pixel coordinates $\left(u,v\right)$ of the AR marker center point can be calculated using the following formula:

(13) Zcuv1=MTecTbeTmb0001

In the above formula, M is the inherent property of the camera, Tec is the hand–eye conversion matrix to be calibrated, Tbe can be calculated by the forward kinematics equation of the manipulator, and Tmb is known and fixed.

Because the translation part of the homogeneous transformation matrix Tmb reflects the coordinates of the AR marker center point in the base coordinate system of the manipulator, the function of Equation (13) is to remap the coordinates of the AR marker center point into the pixel coordinate system. For a certain position $P_{i},i\in N$, the manipulator moves during the calibration process, the AR mark image captured by the camera is denoted as ${img}_{I}$, and the pixel coordinate after the reprojection of the AR marker center point in ${img}_{i}$ is denoted as $q_{i}={\left[u_{i},v_{i}\right]}^{T}$, and then:

(14) qi=ui,viT=projTec,Tibe,Tmb ,i∈N

Because the real pixel coordinates $Q_{i}={\left[U_{i},V_{i}\right]}^{T}$ of the AR marker center point in ${img}_{I}$ can be obtained by corner detection or manual labelling, the reprojection error ${err_{proj}}_{i}$ corresponding to ${img}_{i}$ can be defined as the Euclidean distance between the real pixel coordinates of the AR marker center point and the reprojection coordinates:

(15)  errproji=Qi−qi2=Ui,ViT−projTec,Tbei,Tmb2, i∈N

If the manipulator moves to N positions during the hand–eye calibration process, the average reprojection error can be defined as

(16) $${err\_proj}_{avg}=\sum_{i=1}^{N}{err\_proj}_{i}/N$$

**Figure 7:**
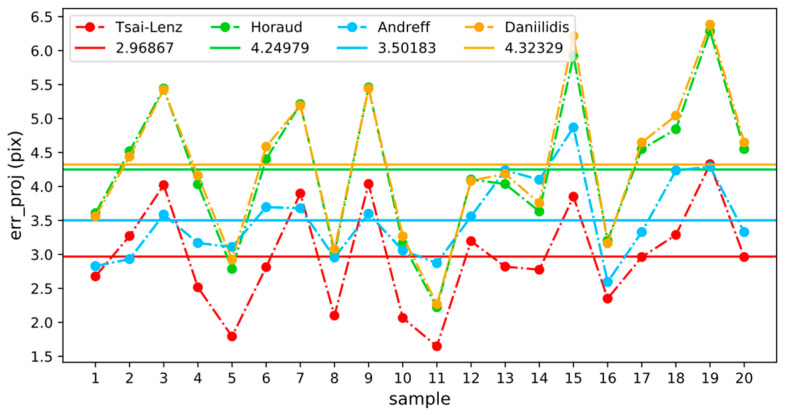
Reprojection error comparison (simulation environment). The average reprojection error associated with the computations from the Tsai–Lenz and Andreff algorithms is minimal, and the variation in the reprojection error for each dataset is relatively modest. This observation aligns with the earlier analyses depicted in Figure 4.

According to Equations (15) and (16), the reprojection error of each group of simulation experimental data was calculated. The results are shown in Figure 7. The horizontal line in the figure represents the average reprojection error of each calibration algorithm. The average reprojection error corresponding to the calculation results of the Tsai–Lenz and Andreff algorithms was small, and the fluctuation in the reprojection error of each group of data was relatively small. In addition, from the previous analysis results, the Euclidean distance errors of these two algorithms were relatively small, proving that the reprojection error reflects the accuracy of the calibration results to a certain extent. Generally, the smaller the reprojection error, the higher the accuracy of the calibration results. Because the real value of the hand–eye transformation matrix cannot be obtained in the process of the hand–eye calibration of a real manipulator, the Euclidean distance error of the calibration results cannot be calculated, and the reprojection error can be used as the evaluation standard for calibration accuracy.

### 3.4. Optimizing Calibration Algorithm by Minimizing Reprojection Error Analysis

From the statistical results of the Euclidean distance error of each algorithm in Figure 1, even if the hand–eye calibration is carried out in the simulation environment, the translation error of the hand–eye conversion matrix calculated by different calibration algorithms is also quite different. Both are greater than 2 mm, which indicates that each calibration algorithm still has a large optimization space.

In the process of hand–eye calibration in a simulation environment, the only error source is the pose calculation error of the AR marker. However, when the above four common algorithms are used for calibration, the calculated Tmc is considered error-free, resulting in a certain error in the calibration results of each algorithm. In other words, the conventional hand–eye calibration algorithm pays more attention to versatility and does not use prior knowledge that the position of the AR marker is fixed in the calibration process; therefore, it is difficult to obtain high-precision calibration results. The definition of the reprojection error of the hand–eye calibration results makes full use of this prior knowledge. According to the previous analysis, the smaller the average reprojection error, the higher the accuracy of the calibration results by minimizing the reprojection error.

**Figure 8:**
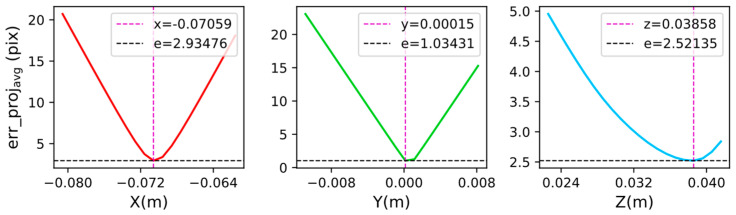
Change curves of reprojection error when adjusting x, y, and z, respectively.

Based on the above analysis, the following exploratory experiments were carried out by controlling the variables to test whether smaller average reprojection errors can be obtained by adjusting the parameters in the calibrated hand–eye transformation matrix. In the experiment, the Tmb used to calculate the reprojection error takes the real values in Table 2, and the translation parameters calibrated by the Tsai–Lenz algorithm in Table 3 are taken as the initial values. The three translation parameters x, y, and z were adjusted with a step length of 0.001 m, and the adjusted hand–eye transformation matrix was substituted into Equations (15) and (16) to calculate the average reprojection error for each group of samples in the simulation experiment. The step size is selected based on the order of magnitude of the translation matrix. If the step size is too large, the search may be too fast, and the optimal solution may be missed. When the step size is too small, it may cause increased computational overhead, especially in high-dimensional parameter spaces. The experimental results are shown in Figure 8. The purple dotted line represents the parameter value of the minimum point, whereas the black dotted line represents the average reprojection error of the minimum point.

As shown in Figure 8, the average reprojection error can be reduced to a certain extent by adjusting the translation parameters separately. The translation parameters calibrated by the Tsai–Lenz algorithm are used as the initial values, which can reduce the search space of the parameters and can help determine the translation parameters corresponding to the lowest point of the reprojection error. However, when z= 0.03858 m, the average reprojection error was minimized; however, this value deviated from the real value $z_{r}=$ 0.0345 m (Table 3). Therefore, translation parameters with higher accuracy cannot be guaranteed by adjusting x, y, and z alone.

**Figure 9:**
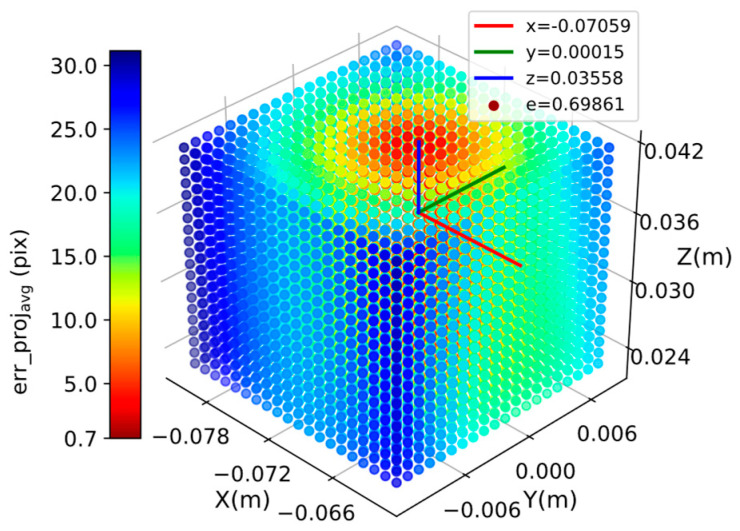
Variation of reprojection error when adjusting x, y, and z parameters simultaneously.

Next, we used 0.001 m as the step length and simultaneously adjusted the x, y, and z parameters. The change rule for the reprojection error is shown in Figure 9. The colors of the data points in the figure reflect the size of the reprojection errors. The translation matrix that minimizes the average reprojection error is $t_{m}={\;\left[-0.07059,0.00015,0.03558\right]}^{T}$, and the corresponding average reprojection error ${err_{proj}}_{avg}=$ 0.69861. The corresponding translation error, ${err}_{t}=$ 0.00165, was calculated using Equation (8). Figure 5 and Figure 7 show that the translation error, ${err}_{t}=$ 0.0022, and the average reprojection, error ${err_{proj}}_{avg}=$ 2.96867, of the calibration results were calculated using the Tsai–Lenz algorithm. There is a set of translation parameters, $t_{b}=\left(x_{b},y_{b},z_{b}\right)$, that can minimize the average reprojection error, and the translation error of this set of parameters, ${err}_{t}$, is less than that of the parameters calibrated using the Tsai–Lenz algorithm. In other words, the accuracy of the hand–eye calibration results can be improved by simultaneously adjusting the three parameters x, y, and z to minimize the reprojection error.

During the calibration process, after a certain position $P_{i}$ of the manipulator is determined, the four parameters Ui,Vi,Tbei,Tmb in Equation (15) are determined accordingly. Therefore, Tec=Tce−1 determines the size of the reprojection error $err_{proj_{i}}$. According to Equation (16), if N positions are determined, then the magnitude of the average reprojection error, $err_{proj_{avg}}$, is uniquely determined by T,ce and the mapping function of the hand–eye transformation matrix, Tce, to the average reprojection error, $err_{proj_{avg}}$, can be defined as

(17) err_projavg=fTce

Furthermore, Tce can be uniquely determined by the translation parameters $t=\left(x,y,z\right)$ and the rotation parameter $r=\left(roll,pitch,yaw\right)$; hence, the mapping function F of the translation and rotation parameters to the average reprojection error, $err_{proj_{avg}}$, can be defined as follows:

(18) $${err\_proj}_{avg}=F\left(t,r\right)=F\left(x,y,z,roll,pitch,yaw\right)$$

Based on the above definition, this study transforms the optimization problem of the hand–eye conversion matrix into the problem of finding the minimum value of the objective function F and optimizes the hand–eye conversion matrix from the perspective of minimizing the reprojection error.

Next, with a step size of 0.0001 m, the three parameters x,y, and z were adjusted to search for the translation parameter that minimizes the function F. The optimal translation parameter $t_{b}=$ (−0.07058, 0.00039, 0.03483) and the corresponding average reprojection error, ${err_{proj}}_{avg}$, is 0.36084, which is 87.845% lower than that of the Tsai–Lenz algorithm. The translation error, ${err}_{t}$, is 0.0007836 m, which is 64.382% lower than that of the Tsai–Lenz algorithm.

In the above optimization process, only the translation parameters in the hand–eye conversion matrix were adjusted. After numerous simulation calibration experiments, it was deduced that the translation parameters had a greater influence on the accuracy of the calibration results than the rotation parameters, and the number of translation parameters was lower. Generally, adjusting only the translation parameters yields ideal calibration results. If the translation and rotation parameters are adjusted simultaneously, higher-precision calibration results can be obtained theoretically, but this process is highly time consuming.

## 4. Hand–Eye Calibration Algorithm Experiment

### 4.1. Calibration Process and Results

To realize the real-time calculation of the spatial posture of the end of the robotic arm, the standard DH parameter method is used to carry out the kinematic modeling of the 7-degree-of-freedom robotic arm in this paper, and the robotic arm link coordinate system is established as shown in Figure 3. The corresponding DH parameters are listed in Table 4. In the table, i represents the joint index, $\alpha_{i-1}$ denotes the rotation angle of link i relative to link i−1, $a_{i-1}$ is the length of the previous link, $d_{i}$ indicates the offset distance of the i-th joint, and $\theta_{i}$ represents the rotational angle of each joint. Specifically, $d_{bs}=0.3705\;m$, $d_{se}=0.3300\;m$, $d_{ew}=0.3200\;m$, and $d_{wf}=0.2255\;m$. In the experimental environment, the size of the AR mark was 10 cm × 10 cm. The RGB camera internal parameters are shown below:

(19) $$\left[f_{x},f_{y},u_{0},v_{0}\right]=\left[1373.72,1374.10,965.286,554.510\right]$$

(20) $$\;\left[k_{1},k_{2},p_{1},p_{2}\right]=\left[0.142,-0.288,\;0.002,\;0.000\right]$$

**Figure 10:**
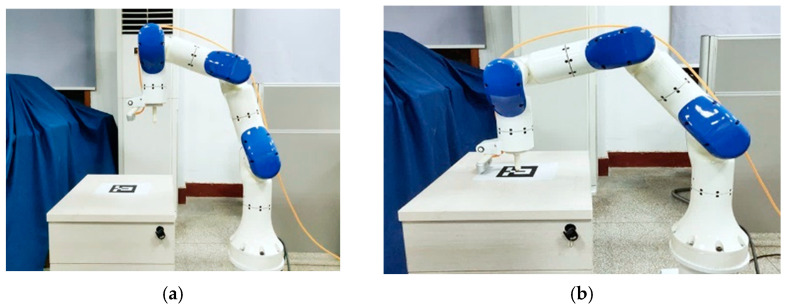
Hand–eye calibration experiment configuration and AR marker center point acquisition. (**a**) Hand–eye calibration experiment configuration. (**b**) AR marker center point acquisition.

Under the premise that the internal parameters of the camera have been calibrated, the hand–eye calibration experiment is performed in a real environment. The experimental configuration is shown in Figure 10a, and the AR mark is placed on the workbench in front of the robotic arm. To obtain the real position of the AR marker, an auxiliary calibration tool is installed at the end of the manipulator, and then the manipulator is manually controlled to align the top of the calibration tool with the center point of the AR marker (as shown in Figure 10b). Finally, the forward kinematics equation of the manipulator is used to calculate the translation matrix of the center point of the AR marker relative to the base coordinate system of the manipulator. The translation matrix is $t_{r}={\left[\begin{array}{ccc} 0.53514\;m & 0.00406\;m & 0.25409\;m \end{array}\right]}^{T}$.

According to the experimental and error analysis results in the simulation environment, the optimized hand–eye calibration process is as follows.

- The real translation matrix $t_{r}$ of the AR marker coordinate system relative to the base coordinate system of the manipulator was obtained using the auxiliary calibration tool, and the position of the AR marker remained unchanged.
- The manipulator was controlled to move to 20 different states where the corner information of the AR marker could be detected, and the corresponding 20 groups of coordinate system transformation data were collected and recorded.
- The mean value Tmb avg of Tmb  is calculated using the coordinate transformation data of each group, and the translation matrix in Tmb avg is replaced by $t_{r}$ to obtain Tmb proj for calculating the reprojection error.
- The Tsai–Lenz algorithm is used to calculate the initial value of the hand–eye transformation matrix Tc e init, and its translation parameters are automatically adjusted to minimize the average reprojection error. The optimized hand–eye transformation matrix is  Tc e optimized.

Because it is difficult to obtain the true value of the rotation parameter in Tmb in a real environment, the average value was used when calculating the reprojection error in the above calibration process. According to the above process, a hand–eye calibration experiment was carried out in a real environment, and the hand–eye conversion matrix Tce was calculated using the four traditional algorithms and the optimized algorithms mentioned above. The results are summarized in Table 4. In addition, in terms of algorithm calibration efficiency, because the calibration formula includes four common arithmetic and matrix operations, there is no requirement for computing power. With the current mainstream CPU (i7 10750H), the calibration time does not exceed 1 ms.

### 4.2. Reprojection Error Analysis

**Figure 11:**
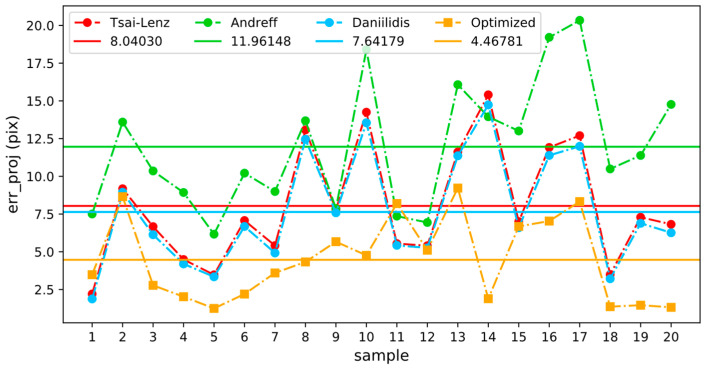
Comparison of reprojection errors. In the real-world context, excluding the Andreff algorithm, traditional algorithms demonstrate similar performance levels. Notably, the optimized algorithm stands out with a significant 44.43% reduction in the average reprojection error compared to the Tsai–Lenz algorithm, showcasing its enhanced efficacy in practical scenarios.

To evaluate the performance difference between the traditional algorithm and the optimized algorithm in a real environment, the reprojection errors corresponding to each hand–eye transformation matrix in Table 4 were calculated using the coordinate transformation data of each group. The results are shown in Figure 11, where the horizontal line indicates the average reprojection error for each method. Because the difference between the hand and eye conversion matrices calculated by the Tsai–Lenz and Horaud algorithms is very small, only the reprojection error corresponding to the Tsai–Lenz algorithm is shown in the figure. In a real environment, except for the Andreff algorithm, the performances of the other traditional algorithms are similar. It is worth mentioning that the average reprojection error of the optimized algorithm is reduced by 44.43% compared with the Tsai–Lenz algorithm. Furthermore, the variability observed in the connecting lines between discrete points in the graph served as an indicator of the robustness of the optimization algorithm. An observation of the figure readily reveals that the fluctuation level in the yellow connecting line, representing the optimization algorithm, is notably reduced compared with the other algorithms. This distinct pattern underscores the heightened robustness of the optimization algorithm compared with its counterparts.

### 4.3. Visual Positioning Error Analysis

In a real scene, the calibration results of each algorithm are used to test the positioning accuracy of the manipulator’s visual positioning. During the test, the AR marker was moved several times, and the state of the manipulator was adjusted to ensure that the corner information of the AR marker could be detected. Position P_c_ of the center point of the AR marker in the base coordinate system of the manipulator was then calculated using Equation (2). Finally, the manipulator was manually controlled, and an auxiliary calibration tool was used to obtain the real position P_r_ of the AR marker center point.

The positioning accuracy of the manipulator in a real scene was quantitatively evaluated, and the visual positioning error was defined as the two norms of the difference between the calculated value P_c_ and the real value P_r_ of the AR marker center point position.

**Figure 12:**
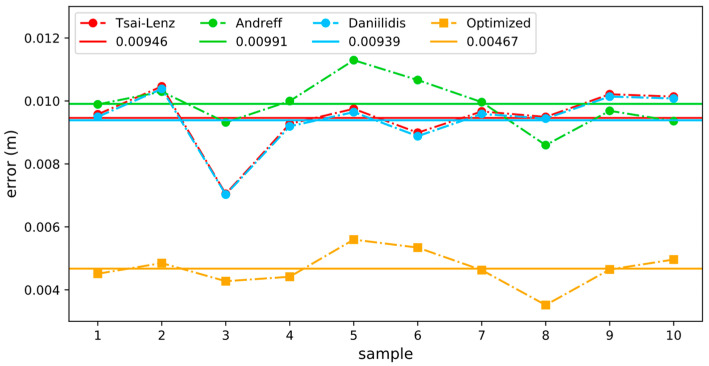
Visual positioning error comparison. The optimized hand–eye calibration method demonstrated a significant reduction in visual positioning error compared to the traditional Tsai–Lenz algorithm, achieving an impressive 50.63% average error reduction. This highlights the efficacy of the optimized approach in substantially enhancing the manipulator’s visual positioning accuracy in real-world scenarios.

In the actual test process, 10 datasets were collected, and the hand–eye conversion matrix calculated by each algorithm was used for the visual positioning of the manipulator. The error statistics are shown in Figure 12. The horizontal line represents the average visual positioning error for each algorithm. The optimized hand–eye calibration method can significantly reduce the visual positioning error of the manipulator. Compared with the traditional Tsai–Lenz algorithm, the average visual positioning error was reduced by 50.63%.

## 5. Summary

To ensure the precision of visual information obtained by a robot, this study delves into the hand–eye calibration algorithm of the manipulator. Commonly used hand–eye calibration algorithms are tested in a simulated environment, and an error analysis is conducted on the results. Subsequently, the reprojection error of the hand–eye calibration results are defined, and an optimization method for hand–eye calibration is proposed based on the eye-in-hand manipulator. Experimental validation is performed in a real environment using the manipulator visual grasping system. The results indicate a significant enhancement in calibration accuracy. Specifically, compared to the Tsai–Lenz algorithm, the proposed optimization algorithm reduces the average reprojection error by 44.43% and the average visual positioning error by 50.63%. Thus, the proposed optimization method markedly enhances the precision of hand–eye calibration results, elevating the overall performance of hand–eye calibration algorithms.

## Figures and Tables

**Table 1:** DH parameter table of 7-DOF robot arm in this article.

i	$\alpha_{i-1}(deg)$	$a_{i-1}(m)$	$d_{i}(m)$	$\theta_{i}(deg)$	$\theta_{imin}(deg)$	$\theta_{imax}(deg)$
1	−90	0	$d_{bs}$	$\theta_{1}$	−180	180
2	90	0	0	$\theta_{2}$	−110	110
3	90	0	$d_{se}$	$\theta_{3}$	−180	180
4	−90	0	0	$\theta_{4}$	−107	107
5	−90	0	$d_{ew}$	$\theta_{5}$	−180	180
6	90	0	0	$\theta_{6}$	−110	110
7	0	0	$d_{wf}$	$\theta_{7}$	−180	180

**Table 2:** True values of parameters in a simulation environment.

	$Translation\;Matrix\;t_{r}\;(m)$	$Rotation\;Matrix\;R_{r}$	$Eulerian\;Angle\;E_{r}\;(rad)$
Tce	$\left[\begin{array}{c} -0.0700\\ 0.00000\\ 0.03450 \end{array}\right]$	$\left[\begin{array}{ccc} 0 & 1 & 0\\ -1 & 0 & 0\\ 0 & 0 & 1 \end{array}\right]$	$\left[\begin{array}{c} 0\\ 0\\ -\pi /2 \end{array}\right]$
Tmb	$\left[\begin{array}{c} 0.45000\\ 0.00000\\ 0.00100 \end{array}\right]$	$\left[\begin{array}{ccc} 0 & 1 & 0\\ -1 & 0 & 0\\ 0 & 0 & 1 \end{array}\right]$	$\left[\begin{array}{c} \pi /2\\ 0\\ \pi /2 \end{array}\right]$

**Table 3:** Hand–eye transformation matrix calculated by calibration algorithms in a simulation environment.

Calibration Algorithm	Tce Computation	
	Translation Matrix	Rotation Matrix
TsaiLenz [7]	$\left[\begin{array}{c} -0.07058\\ 0.00085\\ 0.00325 \end{array}\right]$	$\left[\begin{array}{ccc} 0.00203 & 0.99999 & 0.00337\\ -0.99999 & 0.00204 & -0.00317\\ -0.00318 & -0.00336 & 0.99999 \end{array}\right]$
Horaud [31]	$\left[\begin{array}{c} -0.07160\\ -0.00116\\ 0.00315 \end{array}\right]$	$\left[\begin{array}{ccc} 0.00190 & 0.99999 & 0.00326\\ -0.99999 & 0.00191 & -0.00272\\ -0.00273 & -0.00326 & 0.99999 \end{array}\right]$
Andreff [25]	$\left[\begin{array}{c} -0.07205\\ -0.00032\\ 0.00343 \end{array}\right]$	$\left[\begin{array}{ccc} 0.00185 & 0.99999 & 0.00323\\ -0.99999 & 0.00186 & -0.00250\\ -0.00251 & -0.00322 & 0.99999 \end{array}\right]$
Daniilidi [32]	$\left[\begin{array}{c} -0.07198\\ -0.00116\\ 0.00315 \end{array}\right]$	$\left[\begin{array}{ccc} 0.00161 & 0.99999 & 0.00371\\ -0.99999 & 0.00162 & -0.00215\\ -0.00216 & -0.00371 & 0.99999 \end{array}\right]$

**Table 4:** Hand–eye transformation matrix from calibration algorithms in a real environment.

Calibration Algorithm	Tce Computation	
	Translation Matrix	Rotation Matrix
TsaiLenz [7]	$\left[\begin{array}{c} -0.03740\\ -0.09599\\ 0.04845 \end{array}\right]$	$\left[\begin{array}{ccc} 0.99981 & -0.01313 & -0.01423\\ 0.01346 & 0.99963 & 0.02330\\ 0.01392 & -0.02348 & 0.99962 \end{array}\right]$
Horaud [31]	$\left[\begin{array}{c} -0.03740\\ -0.09599\\ 0.04845 \end{array}\right]$	$\left[\begin{array}{ccc} 0.99981 & -0.01315 & -0.01422\\ 0.01348 & 0.99963 & 0.02330\\ 0.01391 & -0.02349 & 0.99962 \end{array}\right]$
Andreff [25]	$\left[\begin{array}{c} -0.03658\\ -0.09808\\ 0.06191 \end{array}\right]$	$\left[\begin{array}{ccc} 0.99981 & -0.01258 & -0.01464\\ 0.01292 & 0.99965 & 0.02307\\ 0.01434 & -0.02325 & 0.99962 \end{array}\right]$
Daniilidi [32]	$\left[\begin{array}{c} -0.03746\\ -0.09580\\ 0.04834 \end{array}\right]$	$\left[\begin{array}{ccc} 0.99981 & -0.01179 & -0.01497\\ 0.01214 & 0.99965 & 0.02313\\ 0.01469 & -0.02330 & 0.99962 \end{array}\right]$
Optimized	$\left[\begin{array}{c} -0.03540\\ -0.09349\\ 0.05795 \end{array}\right]$	$\left[\begin{array}{ccc} 0.99981 & -0.01313 & -0.01423\\ 0.01346 & 0.99963 & 0.02330\\ 0.01392 & -0.02348 & 0.99962 \end{array}\right]$

## Data Availability

Data sharing is not applicable to this article.
